# Effectiveness of action observation therapy based on virtual reality technology in the motor rehabilitation of paretic stroke patients: a randomized clinical trial

**DOI:** 10.1186/s12883-022-02640-2

**Published:** 2022-03-22

**Authors:** Antonino Errante, Donatella Saviola, Matteo Cantoni, Katia Iannuzzelli, Settimio Ziccarelli, Fabrizio Togni, Marcello Simonini, Carolina Malchiodi, Debora Bertoni, Maria Grazia Inzaghi, Francesca Bozzetti, Roberto Menozzi, Annamaria Quarenghi, Paola Quarenghi, Daniele Bosone, Leonardo Fogassi, Gian Piero Salvi, Antonio De Tanti

**Affiliations:** 1 Diagnostic Department, Neuroradiology Unit, University Hospital of Parma, Via Volturno 39, 43125 Parma, Italy; 2 Department of Medicine and Surgery, University of Parma, Parma, Italy; 3 Cardinal Ferrari Center, S. Stefano Riabilitazione, Fontanellato, Parma, Italy; 4 Quarenghi Clinical Institute, San Pellegrino Terme, Bergamo, Italy

**Keywords:** Action observation therapy, Virtual reality, Mirror neuron system, Stroke, Motor learning

## Abstract

**Background:**

The rehabilitation of paretic stroke patients uses a wide range of intervention programs to improve the function of impaired upper limb. A new rehabilitative approach, called action observation therapy (AOT) is based on the discovery of mirror neurons and has been used to improve the motor functions of adult stroke patients and children with cerebral palsy. Recently, virtual reality (VR) has provided the potential to increase the frequency and effectiveness of rehabilitation treatment by offering challenging and motivating tasks.

**Methods:**

The purpose of the present project is to design a randomized controlled six-month follow-up trial (RCT) to evaluate whether action observation (AO) added to standard VR (AO + VR) is effective in improving upper limb function in patients with stroke, compared with a control treatment consisting of observation of naturalistic scenes (CO) without any action content, followed by VR training (CO + VR).

**Discussion:**

AO + VR treatment may provide an addition to the rehabilitative interventions currently available for recovery after stroke and could be utilized within standard sensorimotor training or in individualized tele-rehabilitation.

**Trial registration:**

The trial has been prospectively registered on ClinicalTrials.gov. NCT05163210. 17 December 2021.

**Supplementary Information:**

The online version contains supplementary material available at 10.1186/s12883-022-02640-2.

## Background

Stroke is the leading cause of disability among adults, and more than 60% of stroke survivors have motor deficits, particularly those related to the upper limb [1]. Stroke rehabilitation usually involves intensive motor training to promote adaptive plasticity by reducing motor deficits and developing new motor learning strategies [2, 3]. It has recently been proposed that the systematic use of action observation (AO) followed by imitation (known as action observation therapy [AOT]) is an effective way to improve motor function and to promote upper limb recovery in patients with motor disorders [4–7]. During a typical AOT session, a series of daily life actions (e.g., grasping a key and inserting it into a lock) are practiced for two to five weeks, with a frequency of three to five sessions per week. During each rehabilitation session, the patient is instructed by the therapist to observe a specific action performed by an actor, presented in a short video-clip on a monitor, and afterwards to reproduce the previously observed action with the paretic limb. The videos usually present a single motor act observed from different perspectives (e.g., subjective, front, or side view). This therapy is based on the neural model of the mirror neuron system (MNS), originally discovered in the monkey premotor and parietal cortex, which is formed by visuomotor neurons that become active both when a monkey performs a goal-directed motor act and when it simply observes the same or a similar motor act performed by the experimenter [8, 9]. A comparable MNS has been identified in humans using different electrophysiological and neuroimaging techniques [10]. In humans, the two main nodes of the MNS are the inferior parietal lobule (IPL) and the ventral premotor cortex (PMv), plus the caudal part of the inferior frontal gyrus (IFG) [11, 12]. 

In the first clinical trial focusing on, Ertelt and colleagues [13] found a significant improvement, evaluated by clinical functional scales, in adult patients with moderate upper limb motor deficits following the application of 18 sessions of AOT, corroborated by a significant increase in activation of the cortical MNS. This improvement was maintained for at least eight weeks after the conclusion of the treatment. More recently, Franceschini and colleagues [14, 15] performed two randomized controlled observer-blinded trials (RCTs) to evaluate the effectiveness of AOT as an add-on treatment to standard upper-limb rehabilitation in acute stroke patients, in which several measures of motor impairment and functional ability demonstrated an improvement over time. The efficacy of AOT has been confirmed in patients with Parkinson’s disease [16, 17], unilateral cerebral palsy (UCP) [18–22] and post-surgical orthopedic patients [23]. AOT is considered particularly useful for activating the motor system in conditions in which intensive motor training is not feasible because of the severity of the impairment of motor function or due to the presence of pain, inflammation, or muscle fatigue [7].

In recent years, new virtual reality-based (VR) rehabilitation treatments have been introduced in order to present rehabilitation exercises in a more practical and friendly setting [24]. These treatments are generally well accepted by patients because they offer several advantages: relatively low cost (in particular for semi-immersive versions), an engaging environment, the real-time personalization of exercises, greater adaptability to the patient’s clinical features and progress, and the opportunity for patients to record their motor performance and get feedback in real time. Furthermore, VR exercises usually require minimal supervision from a therapist, thereby facilitating home-based rehabilitation [25].

Several studies support the application of VR methods in the rehabilitation of the hemiplegic upper limb in stroke patients [26]. The largest study conducted to date involved 376 patients and demonstrated a positive effect of VR-based rehabilitation [27]. Moreover, recent literature reviews [28] have provided evidence for improvement in upper limb motor function and daily life activity after VR-based training compared with standard interventions. However, clinical evidence based on rigorous RCTs on the effect of the combined use of observation of actions followed by their immediate imitation in a VR environment (AO + VR therapy) is lacking, especially in the case of rehabilitation applied during the chronic phase after a stroke.

The main hypothesis of the study is that, for the recovery of motor function of hemiplegic stroke patients in the chronic phase, a combined rehabilitation treatment (AO + VR therapy) is more effective than a control treatment (control observation – CO) based on the observation of videos without motor content (e.g., environmental natural scenes), followed by the execution of actions in VR (CO + VR control therapy).

In sum, the planned trial will examine the following hypotheses:

- AO + VR is an effective tool for promoting upper limb control in paretic stroke patients, and its effects are greater than CO + VR control treatment.
- Motor performance, cognitive level, and structural brain damage assessed before treatment are correlated to the degree of improvement resulting from the AO + VR intervention.
- The AO + VR intervention results in greater plastic functional changes of the MNS activity compared with the effects of the CO + VR control treatment.

## Methods/Design

### Study design

**Fig. 1:**
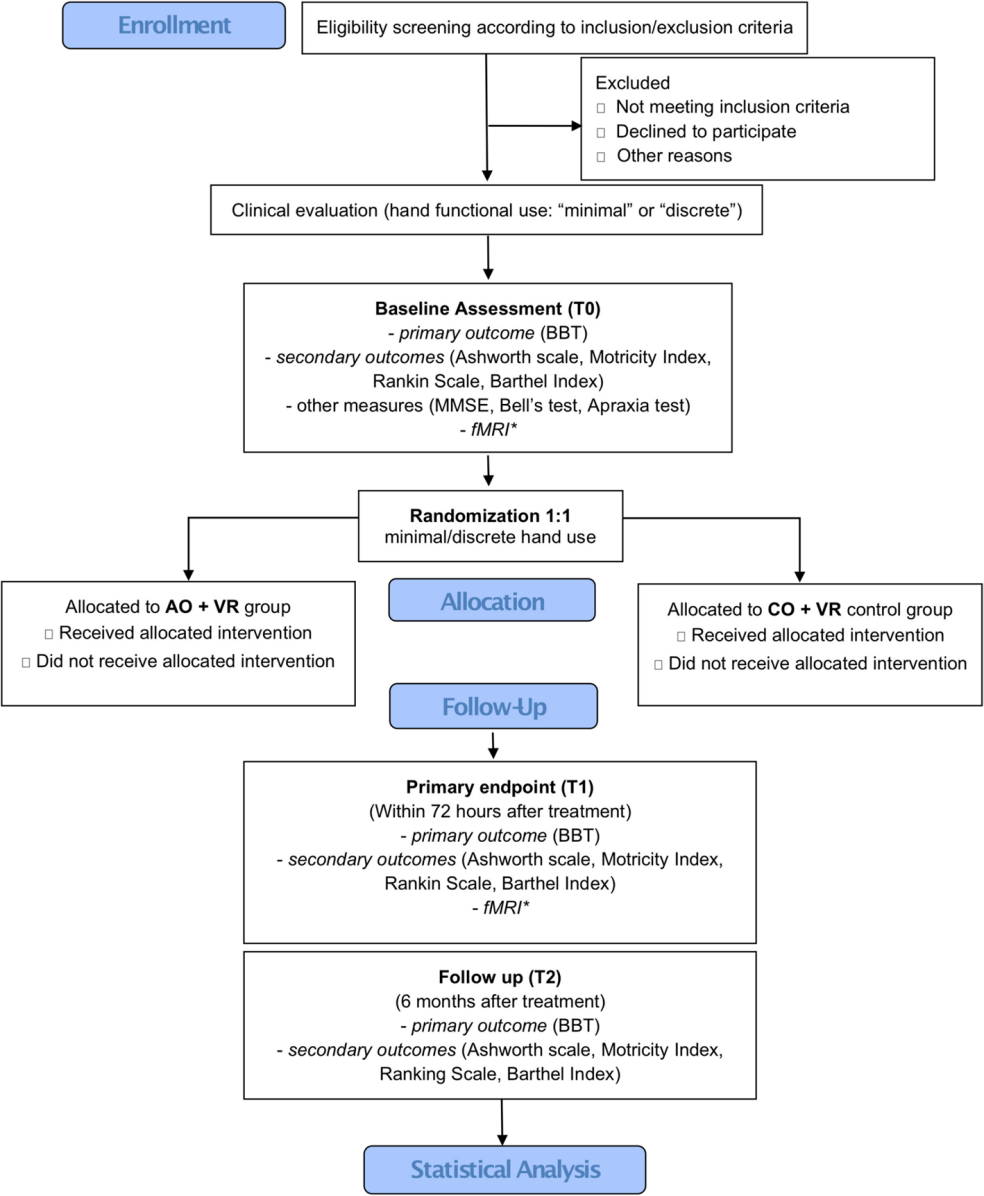
Flow diagram of AOT based on VR study according to CONSORT guidelines. Abbreviations: BBT = Box and Block Test; AO + VR = experimental intervention based on action observation therapy based on virtual reality techniques; CO + VR = control intervention based on observation of control videos followed by the execution of actions using virtual reality

This study will consist in a multicentric randomized allocation concealed (waitlist-controlled) and evaluator-blinded clinical trial (RCT) with two investigative arms, using an intensive rehabilitation program based on AO followed by imitation in VR (AO + VR) (experimental intervention) compared with a control treatment based on observation of control videos (CO) followed by action execution in VR (CO + VR) (control intervention) (see Fig. 1 for a visualization of the flow diagram of the RCT in accord with CONSORT guidelines). This study protocol is in line with the recommendations for clinical trials (SPIRIT) guidelines (Additional file 1).

### Participants

In general, eligible patients will be adults with a clinical diagnosis of stroke (from 3 to 18 months after the acute event, controlled via medical history and patient’s data in the respective rehabilitation center), resulting in primarily motor symptoms with unilateral upper limb paresis and residual movement ability. Only patients with severe motor symptoms or further severe neuropsychological deficits will not be eligible to participate. The multicentric study will take place in two clinical centers representing the main Italian venues for stroke rehabilitation based on VR, i.e., the “Cardinal Ferrari Center” (S. Stefano Riabilitazione) (Parma, IT) and the “Clinical Institute Quarenghi” (Bergamo, IT).

### Inclusion criteria

- a) Primarily motor symptoms with unilateral upper limb paresis (controlled via standard neurological examination).
- b) Residual movement ability of the paretic upper limb, controlled by Medical Research Council (MRC) index (> 2 and < 4), active use of the hemiplegic limb, from minimal (mainly for assistance tasks to the preserved limb) to discrete (characterized by coarse manipulation and an inability to perform precision grip).
- c) Sufficient cooperation and cognitive understanding to participate to the activities, controlled by the investigator recruiting the patient.

### Exclusion criteria

- a) Presence of severe cognitive impairment (score < 20 at Mini Mental State Examination [MMSE]).
- b) Presence of severe forms of unilateral spatial neglect, controlled using the Bells Test (cut-off = / > 50%) [29].
- c) Presence of severe ideomotor Apraxia [30].
- d) Presence of severe anosognosia, assessed by clinical examination.
- e) Presence of severe language comprehension deficits, assessed by clinical examination.
- f) Presence of severe untreated psychiatric disorders.
- g) Sensory impairments hindering participation and/or not compensated visual deficits of central origin.
- h) Drug-resistant epilepsy.

A subgroup of patients will be selected to perform a magnetic resonance imaging (MRI) assessment. For these participants (approximately 10 for each group), additional exclusion criteria will be:

- a) Insufficient cooperation to perform neuroimaging studies lasting approximately 45 min.
- b) Presence of exclusions for 3 T MRI investigations (such as metal implants, prostheses, shunts, etc.). These exclusions will be controlled using an ad hoc questionnaire.

### Interventions

#### Setting for VR rehabilitation

**Fig. 2:**
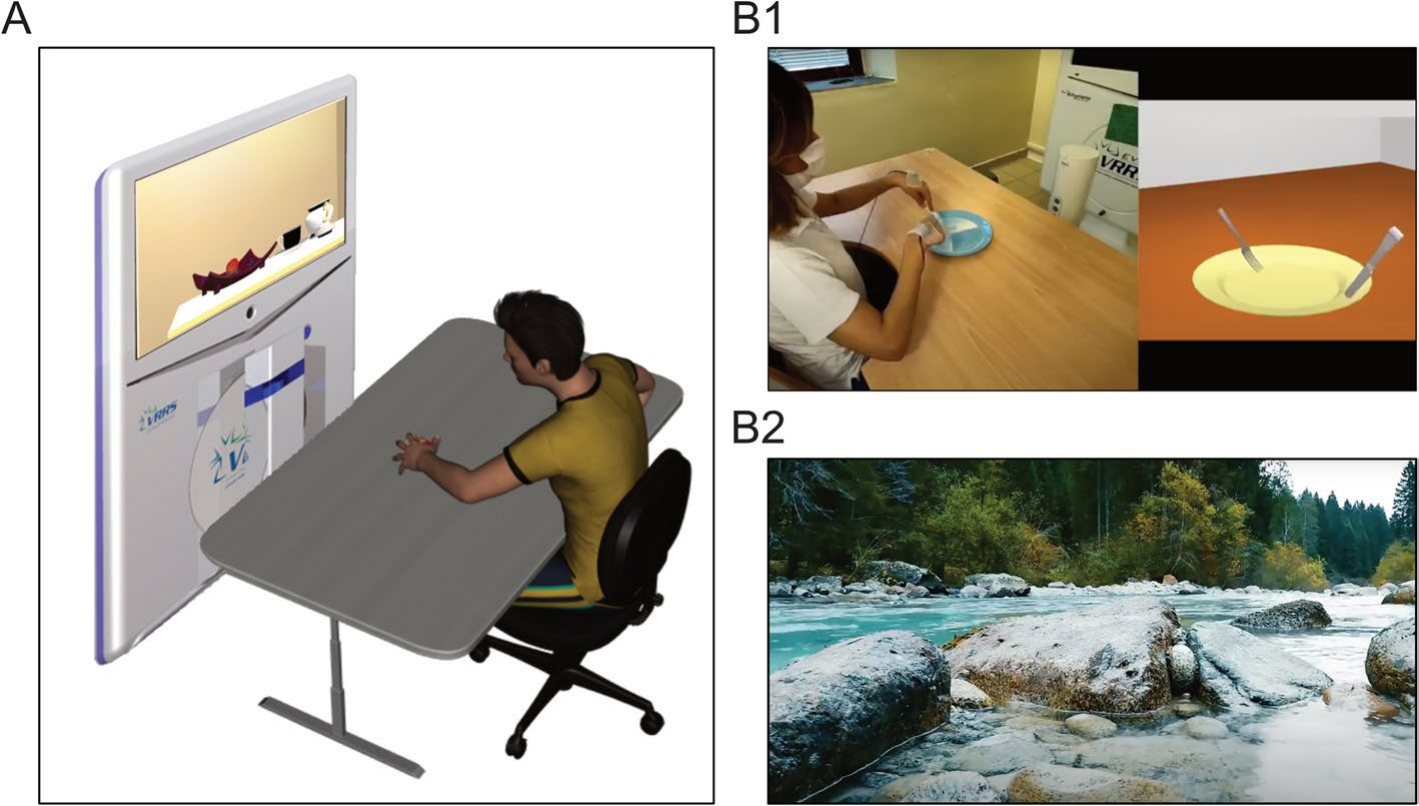
(**A**) Experimental setting for both AO + VR and CO + VR interventions. (**B1**) Static frame showing an example of video-clip to be used for the experimental treatment AO + VR based on observation of actions performed by a model followed by subsequent imitation. (**B2**) Static frame of a video-clip illustrating natural scene, to be used in the CO + VR control treatment

Each rehabilitation session will take place in a quiet room, with the patient sitting on a chair with the arms placed on a table. To provide a suitable range of movement of upper limbs, the patient’s chair will be adjusted so that the table will be at waist height (Fig. 2-A). A large monitor screen (32 inches) will be positioned 1.5 m distance in front of the patient. A trained occupational therapist (OT) or a physiotherapist (PT) will assist the participants, instructing them to pay attention to the video-clips and encouraging them during the imitation task. The Virtual Reality Rehabilitation System (VRRS, Khymeia Group, Noventa Padovana, Italy) will be used for the execution of all exercises in a VR setting. The VRRS is used as a clinical routine for the rehabilitation of a wide spectrum of diseases through the numerous rehabilitation modules containing a large library of clinically validated exercises. The system includes also a workstation connected to a 3D motion tracking system (Polhemus Liberty TM, Colchester, VT) and an integrated high-resolution monitor for the visualization of virtual scenarios.

#### *Experimental treatment (AO* + *VR)*

Participants of the experimental group will undergo a treatment based on observation of actions followed by their immediate reproduction in VR (AO + VR treatment). The experimental treatment will consist in 18 sessions lasting about 1 h each, with a tolerance of ± 2 sessions, thus resulting in a minimum of 16 and a maximum of 20 sessions. Treatment sessions will be carried out with a frequency of about four days per week, within a period of five weeks. During the rehabilitation sessions, the patient will be instructed to carefully watch videos lasting about 1.5 min, presented on the monitor, consisting in unimanual or bimanual actions performed by an actor, from a lateral perspective (see Fig. 2-B1). Subsequently, the patient will be asked to imitate the actions presented for at least three consecutive times, within a time window of 3 min., using the same objects observed in the video, in a virtual scenario (VR), through the Khymeia VRRS system. During the execution of all exercises, the patient will wear also kinematic sensors for three-dimensional tracking of the performed movement, aimed to control objects in the virtual scenario. The treatment will include a total of 52 exercises, arranged as simple movements and complex sequences, either unimanual or bimanual, of increasing complexity. Examples of the proposed exercises are reported in Additional file 2. The protocol will include, for each week of training, a set of exercises subdivided in two parts, the first to be performed for two days, and the remaining to be performed in the remaining two days. For each exercise, the therapist will set the facilitation level, adjusting the sensitivity of kinematic sensors (levels range from 1 to 10) according to the patient's manual impairment. Facilitation level will be used particularly for those patients showing minimal residual hand ability, allowing them to perform the VR exercises. In more details, the exercises during the first week will include “no-gravity” movements (with no hand lifting) such as sliding to reach for a target located in different positions. The second and the third weeks will consists of “simple” and “complex” anti-gravity actions (requiring arm/hand lifting) respectively, either unimanual or bimanual. Furthermore, some bimanual functional exercises will be introduced, such as to put the toothbrushes in a glass, or to use knife and fork. The fourth week will consist of complex bimanual daily life actions, such as, for example, to wear sunglasses, or to draw a line with the ruler. Finally, during the fifth week, a set of more complex anti-gravity exercises will be proposed, including tasks of obstacles avoiding or following a path using a graspable floating pad device. Each session of AO + VR rehabilitation will take 30–45 min. to complete, including changing the setting between different exercises. The administration of personalized set of exercises will be adapted based on the patient hand motor performance at baseline, thus patients with minimal hand and arm ability will perform only no-gravity movements and simple anti-gravity actions, while patients with discrete hand ability will also perform the advanced exercises. Despite this type of adjustment of the treatment all the recruited patients will receive an equal number of treatment sessions, thus the final dose of the treatment will be balanced between the experimental and the control group.

#### *Control treatment (CO* + *VR)*

Participants randomly assigned to the control group will receive an equal number of rehabilitation sessions, as the experimental group. Differently from the latter, patients of the control group will be required to observe videos depicting naturalistic scenes (Fig. 2-B2), without motor contents, for 1.5 min. Then, they will receive a motor training in the VR environment, performing the same type of exercises included in the above-described experimental treatment, prompted by the verbal instructions of an expert therapist. Thus, the general setting for carrying out the rehabilitation sessions will be identical to that of the experimental group, except for the fact that the control group will not be involved in action observation before preforming the exercises. Thus, the control treatment is not based on action imitation, but on purely motor execution.

### Modifications

Temporary suspension of the treatment will be recommended if participants will not be able to perform the VR exercises. The trial will then restart as soon as the patient will be able to participate in the activities. The final decision on the possibility to terminate the trial will belong to the principal investigator. Any adverse event will also be reported using a specific form.

### Adherence reminder sessions

Face-to-face adherence reminder sessions will take place at the beginning of the treatment and after the conclusion of its first part. During these sessions, a member of the staff will provide participants with all instructions about the treatment procedures. In addition, these sessions will be used to reinforce the adherence to the rehabilitation sessions.

### Concomitant care

Participants will continue to take medications for other conditions and rehabilitation treatments as usual care.

#### Monitor committee

Monitoring activity will be guaranteed by the Clinical Trial Quality Team (CTQT) of the University Hospital of Parma (Parma, Italy).

### Outcomes

#### Primary outcome

The primary outcome measure will be the Box and Block Test (BBT) [31, 32], a timed test for assessing upper limb dexterity and motor coordination. The test consists of 150 small wooden cubes (25 mm side) contained in a wooden box. The assessment will be performed by an investigator blinded to the assignment of the patient to the experimental or control group, before treatment (T0), after the conclusion of the treatment (T1, within 72 h of conclusion) and after 6 months, as a follow-up (T2).

#### Secondary outcomes

The secondary outcome measures will include improvement in motor function indices and quality of life measures, i.e., modified Ashworth scale (MAS) [33, 34], Motricity Index [35], Rankin Scale (RS) [36], modified Barthel Index (mBI) [37]. These tests will be administered at both T0 and T1. A further assessment will be performed 6 months (T2) after the end of the treatment to evaluate the retention of the effect.

#### Functional MRI study

A subgroup of participants (approximately 20 individuals equally distributed in the two groups, i.e., 10 for experimental and 10 for control group) will be involved in a functional MRI (fMRI) study, to assess plastic changes in the MNS activity following the application of the rehabilitation treatments. fMRI will be performed in two sessions, one before (T0) and one after the end of the treatment (T1) within 72 h. Before starting each functional imaging session, participants will undergo a training session lasting 15 min, in order to allow them to familiarize with the MR system and the experimental procedure. After, patients will be invited to participate in the real fMRI session (divided into 4 short runs lasting approximately 7 min each).

The patient will be instructed to perform two experimental tasks: a) *action observation* (functional runs 1–2); b) *motor task* (functional runs 3–4).

#### Action observation

During the first two functional runs, visual stimuli will be presented in binocular vision by means of Liquid Cristal Display (LCD) goggles (VisuaStim-SVGA-Resonance Technology, USA). Participants will observe short video-clips (lasting 4 s each), showing unimanual or bimanual actions performed by an actor, from a subjective perspective. The observed actions will consist in grasping and using different tools (e.g., hammering, using a screwdriver, opening a jar etc.), or as control condition, observation of the static initial frame of each clip. The objects will be of different colors in order to increase the visual variability of the stimuli. The visual characteristics of each video will be balanced between the experimental conditions to control the effects of brightness, contrast, sharpness and the amount of visual information. A total of 72 video clips will be created (9 objects × 4 colors × 2 conditions).

Each experimental and control condition will be presented 72 times during the experiment, 36 times for each session. The videos will be presented in independent blocks (*N* = 12) for each condition and each block will contain 6 videos, interspersed with an inter-stimulus period of 800 ms. The duration of each block will be 28 s. A number of 12 blocks will be acquired in each observation run, 6 per condition, followed by a variable *rest* period, lasting 16-20 s. During the *rest* period, in the absence of experimental stimuli, the patients will have to fixate a white cross on a black background.

#### Motor task

In the functional runs 3 and 4, participants will be instructed to perform a motor task consisting in tool manipulation performed with the paretic hand or with both hands. The task will be arranged as for the previous sessions using a block design (block duration: 26 s), in which each block will include 3 motor trials, interspersed with *rest* periods (duration: 16-20 s). During *rest* the patient will simply remain still, fixating the experimental setting, i.e., the own hands and the tool mounted on the apparatus. As control conditions, in separate blocks, participants will perform a simple motor task, consisting in reaching a tool without manipulating it (3 trials per block). Each motor trial of the experimental and control conditions will be presented 30 times for each run (10 blocks per run for each condition). Thus, the entire motor session will consist of 40 blocks, divided in 20 tool manipulation blocks and 20 control reaching blocks.

#### MRI data acquisition and statistical analysis

Structural and functional images will be acquired using a 3 T General Electric scanner (MR750 Discovery) equipped with a 32-channel receiver head-coil. Functional volumes will be acquired either while participants perform the action observation task and the motor task with the following parameters: forty axial slices of functional images covering the whole-brain acquired using a gradient-echo echo-planar imaging (EPI) pulse sequence. Its acquisition parameters will be: slice thickness = 3 mm plus interslice gap = 0.5 mm, 64 × 64 × 37 matrix with a spatial resolution of 3.5 × 3.5 × 3.5 mm, repetition time (TR) = 2000 ms, echo time (TE) = 30 ms, field of view (FOV) = 205 × 205 mm^2^, flip angle = 90°, in plane resolution = 3.2 × 3.2 mm^2^. A three-dimensional (3D) high-resolution isotropic T1-weighted IR-prepared Fast SPGR sequence (called BRAVO, BRAin VOlume) will be acquired as anatomical reference. Its acquisition parameters will be set as follows: 196 slices, 280 × 280 matrix with a spatial resolution of 1 × 1 × 1 mm, TR = 9700 ms, TE = 4 ms, FOV = 252 × 252 mm; flip angle = 9°. The protocol will comprehend a 3D fast spin-echo (FSE) fluid attenuated inversion recovery (FLAIR) sequence (called CUBE FLAIR). The 3D FLAIR is based on 3D-FSE extended echo train acquisition, an advancement of 3D-FSE extended echo train acquisition, an advancement of 3D-FSE in which refocusing flip angle modulation and 2-dimensional acceleration enables volumetric coverage with high in- and through-plane resolution with reduced RF power deposition. Its acquisition parameters will be set as follows: 196 slices, 280 × 280 matrix with a spatial resolution of 1 × 1 × 1 mm, TR = 7000 ms, TE = 118 ms, Echo Train Length 200 ms; FOV = 256 × 256 mm; matrix size 256/256 mm; acceleration factor 2/2. A Diffusion Tensor Imaging (DTI) sequence will be also included: diffusion gradients (b value = 1000 s/mm^2^) applied in 64 noncollinear directions, 8 measures without diffusion gradients (b = 0 s/mm^2^), 45 axial slices with 3 mm slice thickness, FOV 240 × 296 mm, matrix size 80 × 80 (in-plane resolution, 3.0–3.7), TR = 11,000 ms, TE = 92 ms.

#### Sample size

According to CONSORT guidelines [38, 39], the estimated sample size is based on projected treatment effects on the primary outcome measure, the BBT. Based on the available literature [32], we consider a 7-point change in BBT scores at post-test (T1) as a clinically significant effect. Based on a common standard deviation at post-test equal at 11.3 and a drop-out rate of 10%, we estimate that a sample of 47 subjects for each group (*N* = 94) will have a statistical power of 80% to detect a significant difference between the two measurements.

Concerning the fMRI study, the sample size estimate is based on the calculation of the level of change in the Blood Oxygenation Level Dependent (BOLD) signal detected in a previous fMRI study with an action observation paradigm (number of subjects per group = 10). Significant activation clusters within the MNS, including the PMv and the IPL, were considered for the analysis, using a statistical threshold of *p* < 0.001 with Family Wise Error (FWE) correction for multiple comparisons at the cluster level. The Power Analysis (software: NeuroPower-tool) conducted on this study showed that to obtain a change in the BOLD signal with a significance threshold of *p* < 0.05 and a minimum statistical power of 80% with Bonferroni correction, it is necessary to recruit at least 9 participants per group. However, regardless of the presence or absence of effects at the group level, patients will also be treated as a case-series, so that assessment of BOLD change following treatment will also be performed at the single case level.

#### Recruitment

Patients considered eligible (see Methods section “Inclusion and Exclusion criteria”) will be invited from the “Cardinal Ferrari Center” and the “Clinical Institute Quarenghi” to participate in the trial, explaining them in detail the purposes and methods of the study. Before enrollment, the informed consent (see Additional file 3) will be obtained from patients, or from the caregivers.

### Assignment of intervention

#### Randomization

Participants will be randomly assigned to either control or experimental group with a 1:1 allocation using a computer-generated randomization schedule, stratified by clinical level of the hand use (minimal vs. discrete). In particular, permuted blocks of random sizes will be used. The block sizes will not be disclosed, to ensure concealment. All randomization, sequence generation, and preparation of group allocation materials will be performed by an independent researcher, who has no direct contact with the clinical aspects of the trial.

#### Blinding

Participants and their caregivers will be informed about the study aims and procedures, but they will be blinded to group allocation. In the case the patient asks for the presence of the caregiver, she/he will be seated near the participant but out of her/his view, without interfering during the treatment session. The OT/PT performing the intervention will not be blinded to the group allocation. Outcome assessments will be administered and scored by a member of the staff blinded to group allocation.

#### Data management

Each participating center will send the case report form (CRF) to the data manager and the principal investigator of the study (AE). The file will be encrypted, and password protected. All requests for database changes will be tracked and stored. Neuroimaging data acquired at Parma University Hospital will be transferred via Digital Imaging and Communications in Medicine (DICOM) protocol to the Picture Archive Computed System (PACS) for the management of diagnostic images in digital format and will be available for performing the statistical analysis.

At the beginning of the study, to each patient will be assigned an alphanumeric identification code, which does not allow to directly identify the patient’s name. All data collected during the study, except for demographical ones, will be recorded, processed, and stored together with this code. Only the data manager and authorized staff members will be able to associate this code with the patient’s name.

### Statistical analyses

#### Analysis of primary and secondary outcomes measures

Clinical data will be managed and analyzed using the Statistical Package for Social Sciences (SPSS version 21.0). Descriptive statistics will include standard median, deviation, and interquartile distance calculated for both experimental and control groups. For the evaluation of the primary endpoint, the t-Student test for independent samples will be used. In case of excessive deviation from normality, similar non-parametric test (Mann Whitney) will be used.

The primary analysis will be carried out on all participants enrolled in the study, according to the *intention-to-treat* principle. Changes in motor performance between T0 and the subsequent longitudinal evaluations (T1, T2) will be assessed using the repeated measures Analysis of Variance (rmANOVA) with a mixed design 2 × 3, considering the group as “between subjects” factor and the time point measure (T0, T1, T2) as “within subjects” factor.

Missing data will be treated implementing a linear mixed model (LMM) on a “long” database, avoiding “listwise deletion” (elimination of all information related to the subject with missing data). The estimation of parameters deriving from the mixed model will be compared with that related to the rmANOVA analysis, also to assess the impact of missing data. The analyses related to the secondary endpoints will be carried out by applying the same LMM model. Statistical significance will be set at two-tailed alpha error values of *P* < 0.05.

#### fMRI statistical analysis

All functional volumes will be preprocessed and analyzed using SPM12 software (http://www.fil.ion.ucl.ac.uk/spm), implementing a standard pipeline which includes spatial realignment, slice-timing correction, anatomo-functional coregistration, normalization to the Montreal Neurological Institute (MNI) template and smoothing. At group level, significant brain activations will be assessed using the General Linear Model (GLM). Parametric maps (*t* contrast) of the activation will be calculated in the whole brain for each experimental and control condition. Subsequently, to assess possible differences between subjects, a ROI (Regions of Interest) analysis will be performed in the main MNS areas, extracting the BOLD signal associated with each condition. Student’s t-test will be used to evaluate the significance of BOLD signal differences between conditions. All parametric statistical maps will be corrected for multiple comparisons by applying the Family Wise Error (FWE) method, with a significance level set at *p* < 0.001. In addition to the univariate statistical analysis, advanced analyses including Psychophysiological Interaction and Dynamic Causal Modeling will be performed in order to measure effective functional connectivity changes between MNS areas, following the application of the rehabilitation treatment.

#### Study Status

At the time of the submission of this study protocol, recruitment has not yet started.

#### Dissemination policy

The results of the study will be released to the participating patients and to the general medical and neuroscientific community. Every attempt will be made to reduce the minimum interval between the completion of data collection and the release of the study results. We expect to take about 3 to 4 months to draft the results paper for an appropriate peer-reviewed journal.

## Discussion

The present study protocol describes the background and the design for a randomized controlled six-month follow-up trial (RCT) to evaluate the efficacy of AOT combined with VR techniques in improving upper limb activity in patients with stroke. The main hypothesis is that a VR-based therapeutic intervention associated with the systematic observation of actions performed by a model (AO + VR) could increase motor facilitation and, as a consequence, improve upper limb control and function.

The contribution of this study will be significant because the recovery of upper limb function in patients following a stroke represents a challenge for every rehabilitative team. In fact, the traditional rehabilitative approach has substantial and intrinsic limitations in modifying the clinical history of upper limb motor impairment and functional ability. The proposed research is innovative because it is based on a rigorous scientific design and follows CONSORT guidelines, as recommended by evidence-based medicine. Furthermore, it can produce viable targets for future studies that test the effectiveness of rehabilitative interventions for other motor deficits in neurological patients.

The set of AO + VR exercises created for the RCT are goal-oriented and tailored according to the degree of upper limb impairment. This type of personalized treatment allows the adaptation of the exercises to each patient’s upper limb clinical characteristics by proposing specific movements/actions suitable for the patient’s motor repertoire. A specific set of bimanual exercises comprising daily life actions was also included in the rehabilitation treatment, chosen on the basis of their ecological value. Another important feature of the study is that the combined use of action imitation and a VR environment could be useful in activating the motor system in pathological conditions in which intensive motor training is not feasible due to the severity of the impairment. These types of treatments are generally well accepted by patients because they offer an engaging environment and greater adaptability to the patient’s clinical features and progress, as well as the opportunity for patients to record their motor performance. Furthermore, VR exercises usually require minimal supervision by expert therapists, thereby facilitating individualized home-based tele-rehabilitation.

Concerning the role of MNS plastic reorganization in improving motor learning, lesion localization could represent a limiting factor when the lesion affects the cortical areas associated with MNS activity. Nonetheless, a large network of cortical and subcortical areas may provide multiple compensation mechanisms, facilitating motor re-learning in hemiplegic chronic patients. Thus, imaging studies are crucial to explore the potential of the MNS for rehabilitation and to determine which patients may benefit from specific types of AOT treatments. The fMRI assessment included in this protocol specifically aims to investigate the possible functional changes in the MNS activity of patients treated with AO + VR intervention compared to those treated with the control intervention. In particular, this investigation may allow a direct comparison of the patient’s brain activation during the observation and execution of goal-directed actions, enabling the study, for example, of the lateralization of neural activation and the extension of the activated clusters within the MSN. Also, the degree and lateralization of activation in the ipsilesional/contralesional hemisphere may correlate with improvements in arm/hand motor function parameters assessed through the primary and secondary motor outcome measures. This analysis will make it possible to identify the clinical features of stroke patients who would benefit most from AO + VR treatment, thus strengthening the evidence of its effectiveness in specific patients.

The integrated approach described in the present protocol requires a multi-professional team, to plan well-designed randomized controlled trials with the possibility of correlating measures of functional outcomes, neuropsychological assessment, and biological parameters obtained using brain imaging techniques.

## Supplementary Information

**Additional file 1.** SPIRIT Checklist: Recommended items to address in aclinical trial protocol and related documents.

**Additional file 2.** Examples of exercises proposed for therehabilitation treatment.

**Additional file 3.** Informed consent form.

## Data Availability

The datasets used and/or analyzed during the current study will be available from the corresponding author on reasonable request.

## Abbreviations

AO: Action observation
AOT: Action observation therapy
AO + VR: Experimental treatment based on action observation and virtual reality
BBT: Box and Block Test
BOLD: Blood oxygenation level dependent
CO: Control observation
CO + VR: Control treatment based on observation of control videos and virtual reality
CRF: Case report form
DICOM: Digital Imaging and Communications in Medicine
DTI: Diffusion Tensor Imaging
EPI: Echo-planar imaging
FOV: Field of view
fMRI: Functional magnetic resonance imaging
FEW: Family wise error
GLM: General linear model
IFG: Inferior frontal gyrus
IPL: Inferior parietal lobule
LCD: Liquid cristal display
LMM: Linear mixed model
MAS: Modified Ashworth scale
mBI: Modified Barthel index
MI: Motricity index
MMSE: Mini mental state examination
MNI: Montreal Neurological Institute
MNS: Mirror neuron system
MRC: Medical Research Council
MRI: Magnetic resonance imaging
OT: Occupational therapist
PMv: Ventral premotor cortex
PACS: Picture Archive Computed System
PT: Physiotherapist
rmANOVA: Repeated measures analysis of variance
ROI: Region of interest
RS: Rankin scale
TE: Echo time
TR: Repetition time
UCP: Unilateral cerebral palsy
VR: Virtual reality
VRRS: Virtual Reality Rehabilitation System
