# Supplementary material for: Effectiveness of action observation therapy based on virtual reality technology in the motor rehabilitation of paretic stroke patients: a randomized clinical trial
Source: BMC Neurol. 2022 Mar 22;22:109. doi: 10.1186/s12883-022-02640-2 (PMC8939064; doi:10.1186/s12883-022-02640-2)
Supplement: Supplementary file 2 — Additional file 2. Examples of exercises proposed for therehabilitation treatment. [file 12883_2022_2640_MOESM2_ESM.pdf]

Additional file 2. Examples of exercises proposed for the rehabilitation treatment.

Ex.	**Week 1, simple movements**	
1	Lateral reaching movement	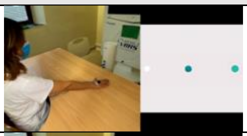
2	Supination/pronation of the hand	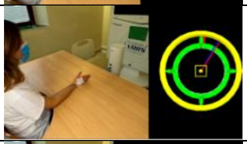
3	Simple trajectory on the horizontal plane	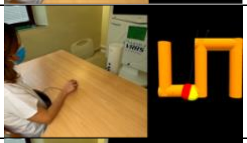
4	Simple trajectory: draw a circle clockwise	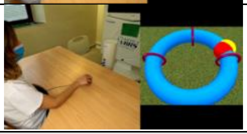
Ex.	**Week 2, simple goal-directed actions**	
1	Grasping a glass and placing it on a shelf	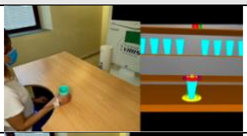
2	Grasping a teapot and move it to the side	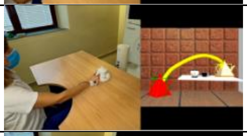
3	Insert the toothbrush in a glass	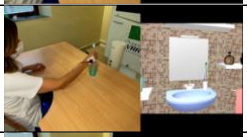
4	Move a book on the chairs with both hands	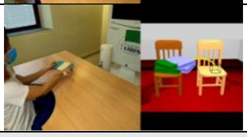
Ex.	**Week 3, antigravity movements**	
1	Grasping an object and moving it towards a target (top-down)	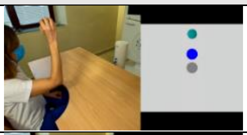
2	Draw a circle with large radius	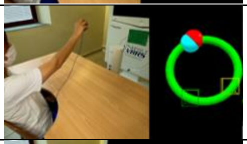
3	Grasp an object and follow a curvilinear path	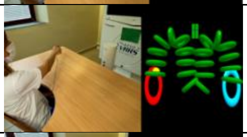
4	Catch water drops with a virtual umbrella	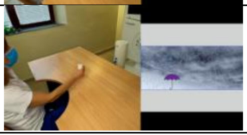

Ex.	**Week 4, daily life actions (functional exercises)**	
1	Wearing glasses (bimanual)	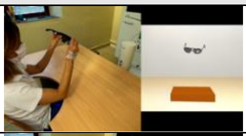
2	Draw a line with the ruler (bimanual)	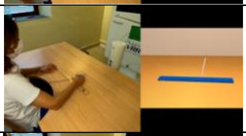
3	Put the toothpaste on the toothbrush (bimanual)	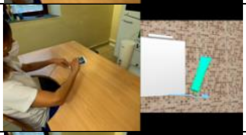
4	Use knife and fork (bimanual)	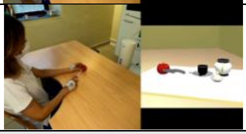
Ex.	**Week 5, complex action sequences with visuospatial components**	
1	Copy a drawing using a graspable pad	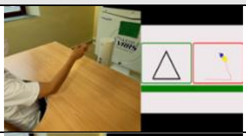
2	Link the points displayed on the screen in the correct order	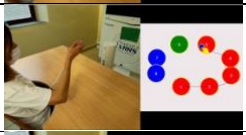
3	Obstacle avoidance: move a ball to the finish without touching the walls	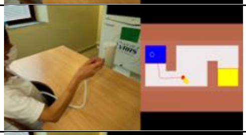
4	Move a ball through the path without leaving it	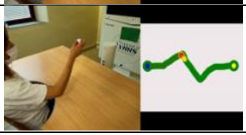
