# Supplementary material for: Effectiveness of action observation therapy based on virtual reality technology in the motor rehabilitation of paretic stroke patients: a randomized clinical trial
Source: BMC Neurol. 2022 Mar 22;22:109. doi: 10.1186/s12883-022-02640-2 (PMC8939064; doi:10.1186/s12883-022-02640-2)
Supplement: Supplementary file 3 — Additional file 3. Informed consent form. [file 12883_2022_2640_MOESM3_ESM.pdf]

Additional file 3. Informed consent form

Title of the trial: "Effectiveness of Action Observation Therapy based on Virtual Reality technology in motor rehabilitation of paretic stroke patients: a randomized clinical trial".

Promoter: University Hospital of Parma, Italy

Sponsor: Italian Ministry of Health (MOH) (Project Code: SG-2019-12370506)

Principal Investigator Name and contact information____

Patient name ____

Date of birth and Place____/___/______

▢ I received from Dr.____ exhaustive explanations about the research question, as reported in the informative form;

▢ I understood the aims, procedures, expected benefits, risks and alternative treatments to the proposed intervention;

▢ I had the opportunity to ask any question to the principal investigator and I received satisfactory answers;

▢ I had enough time to reflect on the received information concerning the study;

▢ I had sufficient time to discuss with others (family, friends etc.) about my participation in the study;

▢ I am aware that the trial protocol and all the forms used have been approved by the Ethics Committee;

▢ I know that the research can be interrupted at any time, by decision of the principal investigator;

▢ I am aware of the importance (and of my responsibility) of informing my family doctor about the trial in which I agree to participate;

▢ I have been informed that the results of the study will be communicated to the scientific community, protecting my identity in agreement with the privacy policy;

▢ I know that any choice expressed in this consent form can be revoked at any time and without any justification;

▢ I received a copy of this consent form;

▢ I agree to participate in the trial;

▢ YES ▢ NOT I wish to be informed of any unexpected findings relating to my own health, which may accidentally result from this study

____ ___/___/______ __________ ________________________
Patient's name Date Signature

____ ___/___/______ __________ __________________________
Legal representant's Name Date Signature
